# Competing Metal–Ligand Interactions in Tris(cyclopentadienyl)-cyclohexylisonitrile Complexes of Trivalent Actinides and Lanthanides

**DOI:** 10.3390/molecules27123811

**Published:** 2022-06-14

**Authors:** Attila Kovács, Christos Apostolidis, Olaf Walter

**Affiliations:** European Commission, Joint Research Centre, Postfach 2340, 76125 Karlsruhe, Germany; christos.apostolidis@web.de

**Keywords:** f elements, actinides, lanthanides, cyclopentadienyl, cyclohexylisonitrile, structure, bonding, XRD, NMR, DFT

## Abstract

The structure and bonding properties of 16 complexes formed by trivalent f elements (M=U, Np, Pu and lanthanides except for Pm and Pr) with cyclopentadienyl (Cp) and cyclohexylisonitrile (C≡NCy) ligands, (Cp)_3_M(C≡NCy), were studied by a joint experimental (XRD, NMR) and theoretical (DFT) analysis. For the large La(III) ion, the bis-adduct (Cp)_3_La(C≡NCy)_2_ could also be synthesized and characterized. The metal–ligand interactions, focusing on the comparison of the actinides and lanthanides as well as on the competition of the two different ligands for M, were elucidated using the Quantum Theory of Atoms in Molecules (QTAIM) and Natural Bond Orbital (NBO) models. The results point to interactions of comparable strengths with the anionic Cp and neutral C≡NCy ligands in the complexes. The structural and bonding properties of the actinide complexes reflect small but characteristic differences with respect to the lanthanide analogues. They include larger ligand-to-metal charge transfers as well as metal–ligand electron-sharing interactions. The most significant experimental marker of these covalent interactions is the C≡N stretching frequency.

## 1. Introduction

The cyclopentadienyl (Cp) ligand belongs to the most popular complexing agents in organometallic chemistry. It has been used for complexing rare earth elements since 1954 [1,2,3]. The first related actinide (An) complex, (Cp)_3_UCl, synthesized by Reynolds and Wilkinson in 1956 [4], was actually the first isolated organouranium compound. In the next two decades, other An(Cp)_3_ complexes with trivalent An from Th to Cf were introduced [5,6,7,8,9,10,11,12,13]. Since then, numerous An cyclopentadienyl derivatives have been synthesized and characterized [3,14,15,16,17,18]. A special application of Cp-derivative ligands is based on their efficient stabilization of the +2 oxidation states of lanthanide (Ln) and middle-row An elements [19].

Tris(cyclopentadienyl) complexes are very suitable models for comparing the bonding of 4f and 5f elements. The high symmetry of these pseudo-*D_3h_* structures is particularly advantageous for a detailed analysis of individual orbitals and to determine their role in the covalent interactions [20,21,22,23,24,25,26]. Thus, early Xα-SW molecular orbital calculations concluded that in (Cp)_3_-complexes light An show larger covalency than Ln and heavier An [22,27]. This larger covalency was attributed to the significant involvement of the 5f orbitals in bonding in the former complexes. A recent, more sophisticated DFT study on the first half of the An row (from Th to Cm) concluded that the An-Cp bonding had a very ionic character, which increased along the An row [25,26]. The covalency in terms of the An-ligand orbital overlap and the electron density in the region between the An and carbon nuclei decreased towards the heavier An. In contrast, the molecular orbital compositions, atomic populations and metal spin densities showed the largest mixing between the An and ligand levels for Pu(Cp)_3_–Cm(Cp)_3_, due to a (coincidental) energy match of the An and ligand orbitals (called an energy-driven covalency). Similar conclusions have been drawn in related studies on An(Cp)_4_ complexes containing a tetravalent An [26,28]. Another noteworthy study on M(Cp)_3_ complexes assessed the bonding of transition metals (TM = Sc and Y), Ln (La, Ce, Yb and Lu) and An (Ac and Th) [29]. Increasing the covalent character of the metal–ligand bond in the order TM > An > Ln was obtained in terms of energy decomposition analysis and the charge transferred from Cp to M.

**Figure 1:**
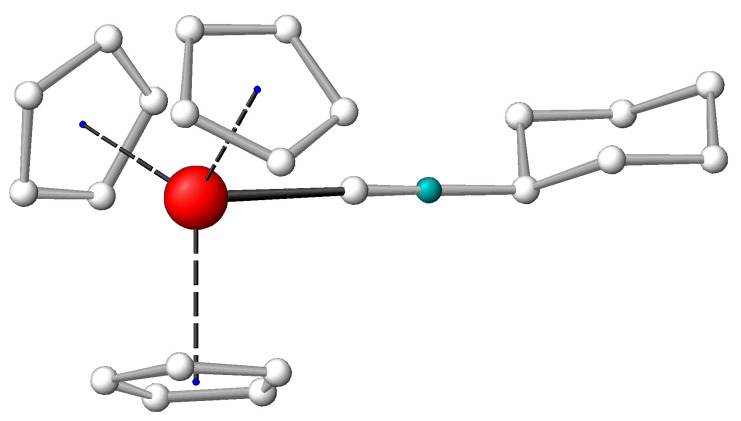
Arrangement of the ligands in the (Cp)_3_M(C≡NCy) complexes. C atom in grey, metal in red, N in blue green and H atoms omitted for reasons of clarity.

A comparison of the cyclohexylisonitrile adducts (Cp)_3_M(C≡NCy) (presented in Figure 1) of An and Ln can provide further insight into the bonding differences of the 4f and 5f elements. The advantage of these compounds is the IR-sensitive C≡N stretching vibration (ν_C≡N_) influenced by the bonding interactions of the C≡N group [7,30]. The accurately measurable vibrational frequency efficiently extends the crystal structure data, which can suffer from experimental uncertainties in the magnitude of the small differences between the various complexes. Combined with quantum chemical modeling, a joint experimental–theoretical analysis offers a straightforward characterization of the molecular (particularly the bonding) properties.

There are limited data on the structure of complexes of f elements with cyclopentadienyl + cyclohexylisonitrile ligands. Only the crystal structure of (Cp)_3_Pr(C≡NCy) has been reported in an early paper [31]. Regarding the derivatives, the structures of five Ln complexes could be found in the CCSD database: (Me_5_Cp)_3_La(C≡NtBu) and (Me_5_Cp)_3_Nd(C≡NtBu) [32], (MeCp)_3_Ce(C≡NtBu) and [(SiMe_3_)_2_Cp]_3_Ce(C≡NtBu) [33] and (tBuCp)_3_Ce(C≡NtBu) [34]. In addition, the crystal structures of three related U complexes have been reported: (Me_3_SiCp)_3_U(C≡NEt) [35], (Me_4_Cp)_3_U(C≡NC_6_H_4_OMe) [36] and (Cp)_3_U(C≡NtBu)(O_3_SCF_3_) [37]. 

In the present study, the (Cp)_3_Ln(C≡NCy) complexes of the entire lanthanide series (except for Pm and the Pr one described in the literature [31]) were synthesized and characterized by X-ray diffraction. From the 5f elements, the related U, Np and Pu complex, (Cp)_3_An(C≡NCy), was synthesized and characterized accordingly. However, the quality of the crystals allowed for an X-ray study of the Pu complex only. The synthesis and IR spectrum of the U complex was published in Ref. [7].

## 2. Results and Discussion

### 2.1. Crystal and Molecular Structure

XRD experiments were performed on the complexes of 13 Ln and Pu. Whereas the crystals of most of the Ln complexes were stable and of excellent quality, facilitating the determination of the structures with good accuracy (exceptions were La and Ce with large experimental uncertainties), the crystal of (Cp)_3_Pu(C≡NCy) decomposed during the measurement. Therefore, for the latter complex, we had to be satisfied with low-quality crystals. The crystals of the corresponding U and Np complexes were of even lower quality, unable to be used for XRD analysis.

**Figure 2:**
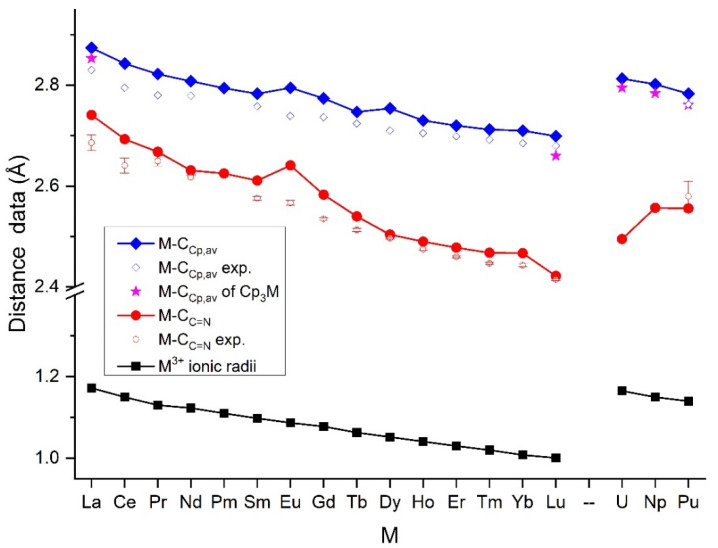
The metal–ligand M-C_C≡N_ and average M-C_Cp_ distances (Å) of (Cp)_3_M(C≡NCy) and M(Cp)_3_ complexes as well as the six-coordinate effective M^3+^ ionic radii [40]. The non-averaged experimental data are extended with the standard deviation bars.

Crystallographic details of the studied complexes are given in Table S1 in the Supplementary Material. From the complexes, only (Cp)_3_La(C≡NCy) was crystallized in the triclinic system; all the others had a monoclinic crystal character. The distances most characteristic of complex formation, viz., M-C_C≡N_, C≡N and the average of the M-C_Cp_ distances from the XRD measurements, are presented in Table 1. They are compared with computed ones in Figure 2. The computed values presented in Figure 2 and additional data (the C≡N bond distances and C≡N stretching frequencies) are given in Table S2 of the Supplementary Material.

**Table 1:** Selected experimental data: bond distances and C≡N stretching frequencies of (Cp)_3_M(C≡NCy) complexes ^1^.

M	M-C_C≡N_	C≡N	M-C_Cp,av_	ν_C≡N_
La	2.686(16)	1.147(6)	2.830	2180 ^3^
La’	2.818(6)	1.149(8)	2.854	-
Ce	2.641(15)	1.145(18)	2.795	2197 ^3^
Pr ^2^	2.65(1)	1.11(1)	2.78	2203 ^3^
Nd	2.618(4)	1.143(5)	2.779	2207 ^4^
Sm	2.576(4)	1.149(5)	2.758	2202 ^3^
Eu	2.567(5)	1.149(5)	2.739	2200 ^3^
Gd	2.535(2)	1.147(3)	2.737	2196 ^3^
Tb	2.513(3)	1.150(3)	2.724	2205 ^4^
Dy	2.497(2)	1.149(2)	2.710	2204 ^3^
Ho	2.475(2)	1.150(2)	2.705	2205 ^4^
Er	2.460(2)	1.147(2)	2.699	2206 ^3^
Tm	2.447(2)	1.148(2)	2.692	2204 ^3^
Yb	2.443(3)	1.155(3)	2.685	2203 ^4^
Lu	2.415(2)	1.149(3)	2.680	2210 ^3^
U	-	-	-	2160 ^5^
Np	-	-	-	2166 ^3^
Pu	2.58(3)	1.10(3)	2.762	2190 ^3^

^1^ Bond distances are given in angstroms with standard deviations in parentheses, whereas the C≡N stretching frequencies are in cm^−1^. La’ refers to the (Cp)_3_La(C≡NCy)_2_ complex; the (close) data of the three conformers were averaged. ^2^ Bond distances from Ref. [31]. ^3^ From Refs. [38,39]. ^4^ From Ref. [30]. ^5^ From Ref. [7].

The metal–ligand distances were determined by the competing bonding interactions of the two types of ligands with the M^3+^ ions. Based on the anionic character of Cp, the M-Cp interactions were predominantly ionic. Accordingly, this interaction was governed by the M^3+^ ionic radii [40] and resulted in a gradual decrease in the metal–ligand bond distances, as observed in numerous studies on series of Ln complexes [41,42,43,44,45,46], specifically those on Ln complexes with Cp-type ligands [47,48].

Figure 2 demonstrates the trends for the Ln-C bond distances in the (Cp)_3_Ln(C≡NCy) complexes; they decreased in accordance with the Ln^3+^ ionic radii. In general, the experiment and computations showed a good agreement, with the computed bond lengths slightly overestimated. The exceptions were the Eu-C_C≡N_ and Eu-C_Cp_ distances of the Eu complex, where the overestimated computed values broke the decreasing trend. This peculiar feature was inspected using other codes (ADF with an all-electron basis set), other DFT functionals (TPSSh) as well as probing for an eventually lower-energy electronic state of this complex by switching the occupied and virtual orbitals, but the contradiction with the experimental trend remained.

The An-ligand distances, in agreement with the larger An^3+^ radii, were larger than the Ln-ligand ones. Similar to the Ln complexes, the An-C_Cp_ distances decreased parallel with the An^3+^ radii, contradicting the reported marginal increase from U(Cp)_3_ to Pu(Cp)_3_ in Ref. [25]. Among the An-C_C≡N_ bond, the U-C_C≡N_ one deviated from the trend of the ionic radii, showing a significant drop. This phenomenon suggests significantly stronger U-(C≡NCy) bonding interactions compared to the other complexes.

In contrast to the Ln-ligand distances, the C≡N bond lengths and stretching frequencies (ν_C≡N_) of the Ln complexes compiled in Table 1 and Table S2 showed no relation to the Ln^3+^ ionic radii. The experimental results scattered slightly around 1.15 Å and 2200 cm^−1^, respectively, whereas the related computed data was around 1.163 Å and 2260 cm^−1^, respectively. The computed bond distance and harmonic frequency of the free C≡NCy ligand was 1.170 Å and 2173 cm^−1^ (experimentally, it was 2136 cm^−1^ [30]), respectively. The slight contraction of the C≡N bond in the complexes with respect to the free ligand was a consequence of the metal–ligand interactions. The C≡N bond was strengthened by coordination, which is typical for the strong σ-donor character of C≡NCy combined with its low π–acceptor abilities. Accordingly, the coordination shifted the stretching vibration of the C≡N bond to higher wavenumbers, as shown by both the experimental and computed results. We note that the experimental C≡N stretching frequency of 2180 cm^−1^ of (Cp)_3_La(C≡NCy) was somewhat lower than that of the other Ln complexes (Table 1). This may rather be attributed to the different crystal structure of the La complex (vide supra) than to interactions with C≡NCy being different from the rest of the Ln row.

The above parameters of the C≡N triple bond were changed characteristically in the (Cp)_3_An(C≡NCy) complexes. The experimental C≡N stretching frequencies reported between 2160–2190 cm^−1^ (An = U, Np, Pu) [7,38] were significantly lower than those of the (Cp)_3_Ln(C≡NCy) complexes (Table 1). The frequency of the (Cp)_3_U(C≡NCy) complex agreed with the one of 2160 cm^−1^ measured for (Me_3_SiCp)_3_U(C≡NEt) [35]. It also compared well to the C≡N stretching frequencies of complexes of the type (CpR)_3_U(C≡NR’) with aliphatic isonitriles coordinating to the U center, reported in the range between 2127 and 2180 cm^−1^ [36,37].

In agreement with the experiment, our DFT computations predicted a significant decrease in the C≡N stretching frequencies in (Cp)_3_An(C≡NCy) compared to those of the (Cp)_3_Ln(C≡NCy) ones (cf. Table S2). The structural reason for this feature is clear from the computed C≡N bond distances being significantly larger than those of the (Cp)_3_Ln(C≡NCy) ones (Table S2). Hence, ν_C≡N_ represents a sensitive and experimentally unambiguously detectable marker of the different bonding properties of the 5f elements with respect to the 4f ones. On this basis, the differences in the bonding of the present three An (U, Np and Pu) could also be validated.

We note that the unexpectedly small XRD value for the C≡N bond of (Cp)_3_Pu(C≡NCy) contradicts its low ν_C≡N_ frequency (cf. Table 1) as well as the above outlined trends. However, due to the mentioned low quality of this sample resulting in large experimental uncertainties in the XRD data, the reliability of this experimental C≡N bond distance is rather limited.

### 2.2. Structure of the (Cp)_3_La(C≡NCy)_2_ Bis-Adduct

**Figure 3:**
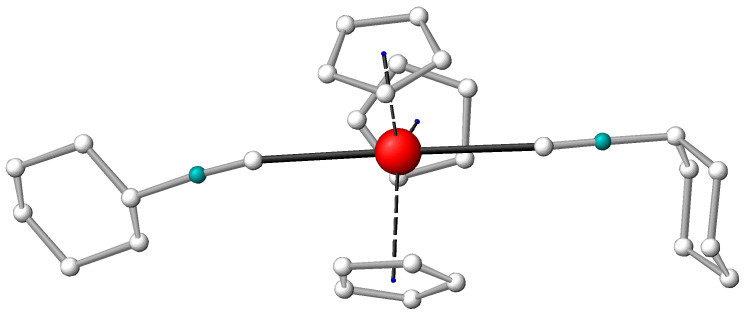
View to the molecular structure of (Cp)_3_La(C≡NCy)_2_ in the solid state; presented is the isomer with one equatorial (on the left) and one axial (on the right) C≡NCy conformer. C atom in grey, metal in red, N in blue green and H atoms omitted for reasons of clarity.

If the free metal site opposite to the C≡NCy ligand is large enough, an additional ligand can coordinate at that site. A few Ln metals (La and Ce) and U were probed for this reaction, but the bis-adduct could be isolated only in the case of the largest Ln(III) ion (La^3+^). The XRD analysis confirmed its constitution as (Cp)_3_La(C≡NCy)_2_. The La metal was in the center of a distorted trigonal bi-pyramidal structure with the two C≡NCy ligands along the vertical axis of the bi-pyramid and the three Cp ligands in the trigonal plane. Interestingly, there were three different molecules in the crystallographically independent unit of the elementary cell, differing in the conformation of the C≡NCy moieties. In one conformer, the C≡N groups were bonded in an axial position to the Cy chair structure, whereas in the second conformer axial–equatorial (as presented in Figure 3) and in the third conformer equatorial–equatorial, arrangements were found (for details on the crystal structure, see Table S1). DFT computations confirmed the close energies of the three conformers (within 10 kJ/mol in the gaseous phase) and supported the measured structural characteristics. The axial C≡N-Cy conformations were stabilized by van der Waals interactions of the two Cy hydrogens with the anionic Cp rings (these computed H_Cy_…H_Cp_ distances were around 2.4 Å, the vdW radius of H being 1.2 Å).

The average La-C_C≡N_, C≡N and La-C_Cp_ distances of the (Cp)_3_La(C≡NCy)_2_ conformers are included in Table 1, while the computed ones are in Table S2. The experiment and theory agreed in the elongation of all the La-C bond distances upon bis-coordination. From the two types of La-ligand bond distances, the La-C_C≡N_ one lengthened more considerably (from 2.68 to 2.81 Å, Table 1), rationalized by a competition of the two trans-positioned C≡NCy ligands for the same acceptor orbitals of La. In contrast, the strong C≡N bond increased only marginally, resulting in a marginal decrease in ν_C≡N_ (cf. Table S2).

An n-pentane extraction of (Cp)_3_La(C≡NCy)_2_ led to the formation of crystals of the constitution (Cp)_3_La(C≡NCy)*(Cp)_3_La(C≡NCy)_2_, where, in one crystal unit cell, both the mono- and the bis-adducts were found (for more details, see Table S1). The metal–ligand distances in the (Cp)_3_La(C≡NCy)*(Cp)_3_La(C≡NCy)_2_ adduct were very close to those of the pure (Cp)_3_La(C≡NCy) and (Cp)_3_La(C≡NCy)_2_ complexes.

### 2.3. ^1^H and ^13^C NMR Characteristics of (Cp)_3_Pu(C≡NCy)

**Figure 4:**
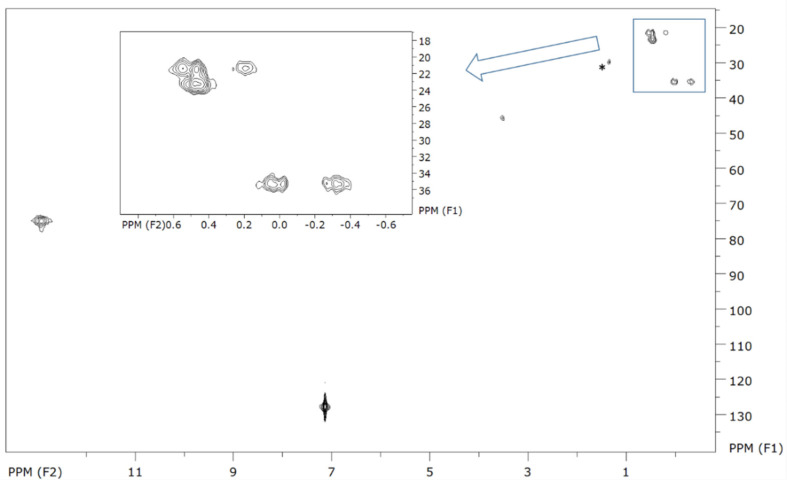
2D CH correlation NMR spectrum of (Cp)_3_Pu(C≡NCy) measured in C_6_D_6_, including all detectable cross peaks. Inset is the magnification of the marked zone. The peak marked with a * arises from decomposition of the sample.

The full assignment of the ^1^H and ^13^C NMR spectra of (Cp)_3_Pu(C≡NCy) required the registration of appropriate 2D HH and CH correlation NMR spectra too (Figure 4 and Figure S3). The assignments are given in Table 2.

**Figure 5:**
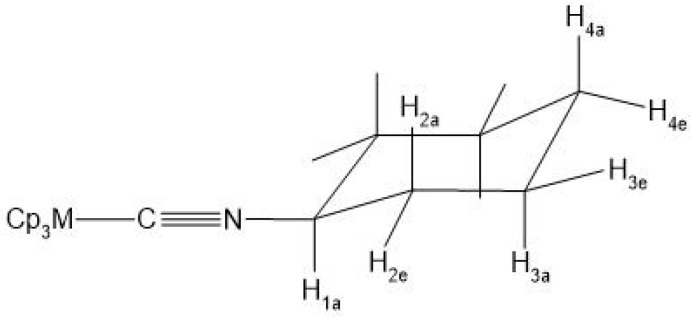
Schematic drawing of (Cp)_3_M(C≡Ncy) with the proton labeling used in the assignment of the NMR signals.

The three Cp rings resulted in one resonance peak at 12.99 ppm in the ^1^H NMR spectrum, in agreement with the values reported in [18,49,50,51], and gave rise to a cross peak at 75.0 ppm in the 2D CH correlation spectrum (Figure 4). This confirmed that the Cp rings were involved in a fast dynamic process. This situation was different for the cyclohexyl (Cy) residue of the isonitrile ligand. It is known to perform (at room temperature with the NMR timescale comparably slow) an interchange between two chair conformers in these complexes. Temperature-dependent ^1^H NMR studies on the (Cp)_3_Pr(C≡NCy) complex in a toluene solution revealed a conformer mixture of 2:1 at 30 °C [52]. The present ^1^H NMR spectrum of (Cp)_3_Pu(C≡Ncy) (Figures S1 and S2) similarly showed a pattern of seven signals raised by 11 protons of the Cy residue. Though the signals were partially overlapping, they could be distinguished and assigned to axial and equatorial protons (Figure 5) by 2D HH and CH correlation NMR spectroscopy.

The major conformer should have the C≡N group in the equatorial position at the Cy ring, as found in the crystal structures (vide supra) and in the case of (Cp)_3_Pr(C≡Ncy) in the solution [52]. The axial proton at C_1_ (H_1a_) had a characteristic downfield chemical shift and gave rise to a resonance peak at 3.56 ppm and a corresponding ^13^C resonance peak at 75.0 ppm. It gave, in the 2D HH correlation spectrum, a cross peak with the axial proton on C_2_ of the Cy ring (H_2a_, Figure S3), the latter represented by a ^1^H resonance peak at −0.29 ppm and a corresponding ^13^C resonance peak at 35.4 ppm (Table 2). Via a 2D CH correlation, the resonance peak at 0.01 ppm in the ^1^H NMR spectrum was assigned to its equatorial pendant H_2e_, which was additionally confirmed by a strong cross peak between them in the 2D HH correlation spectrum. Continuing in this way, the two protons of the C_3_ atom of the Cy ring (with a ^13^C shift of 21.4 ppm) showed their resonance peaks at 0.23 and 0.51 ppm. Due to the symmetry plane in Cy, the peak intensities of the H_2_ and H_3_ protons were two times larger than those of H_1_ and H_4_. The C_4_ atom of the Cy ring could be assigned to the ^13^C resonance peak at 23.2 ppm whereas its two protons could be assigned to the broad 0.46 ppm signal in the ^1^H NMR spectrum. As a result of the above analysis, the (Cp)_3_Pu(C≡Ncy) complex can be added to the scarce examples of organometallic Pu complexes with a complete assignment of the ^1^H and ^13^C NMR spectra [18,50,51].

### 2.4. Bonding Analysis

The (Cp)_3_M(C≡NCy) complexes were formed by an interplay of ionic and covalent interactions. Predominantly ionic interactions were expected between the (formally) M^3+^ and Cp^-^ ions. The QTAIM atomic charges of M were around +1.8 e (Table S3). From the Cp ligands, a considerable charge transfer to M^3+^ (ca. 0.4 e from each Cp) occurred, leaving a net charge of ca. −0.6 e on the Cp. Recent topological analyses of M(Cp)_3_ and M(Cp)_4_ (M = f-element) complexes resulted in small electron densities (ρ(r) around 0.04 au), positive Laplacians of electron density (▽ ρ(r) around 0.10 au) and close to zero total electronic energy densities (H(r) around −0.002 au) at the M-C_CP_ bond critical points (BCP) [25,26,28]. These are characteristic of strongly ionic bonding interactions [53,54,55]. In the present study, very close values of the above topological parameters (around 0.03, 0.09 and −0.001 au, respectively) were found at the M-C_Cp_ BCP-s of the (Cp)_3_M(C≡NCy) complexes. Hence, the introduction of the C≡NCy ligand did not change the strongly ionic character of the M-Cp_3_ bonding, and altogether caused only minor alterations in the above topological parameters.

**Figure 6:**
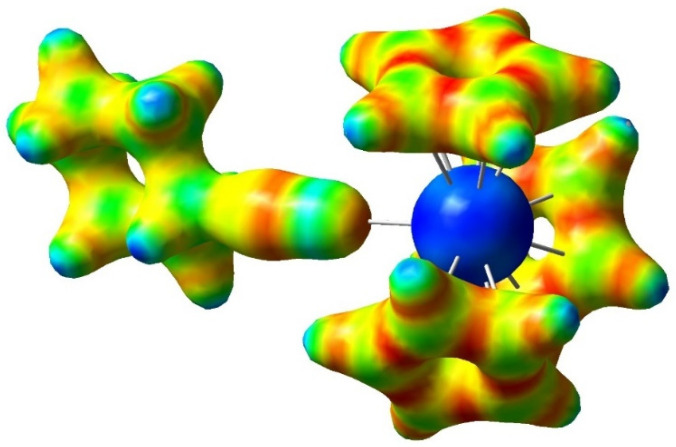
Electrostatic potential (ESP) map of (Cp)_3_La(C≡NCy) with density isovalue of 0.11 au; red—mostly negative and blue—mostly positive.

Summing up the QTAIM atomic charges of the C≡NCy ligand in the present (Cp)_3_M(C≡NCy) complexes, values between +0.04 and −0.08 e were obtained, indicating that the formally neutral charge of the C≡NCy ligand did not change significantly in the complex. On this basis, also taking into account the positive atomic charge of C_CN_ (cf. Table S3), only a small ionic contribution should be expected for the M-(C≡NCy) bonding. The case, however, was more complex. The charge distribution in (Cp)_3_M(C≡NCy) can be followed in the ESP map in Figure 6. The colors were in agreement with a strongly negative character of the N atom and with a slightly positive one of C_C≡N_. However, at the same time, the ESP map also showed that the charge distribution around C_C≡N_ was strongly polarized: as a result of the polarizing effect of the M cation, a significant charge concentration (local negative partial charge) appeared at the side facing the positively charged M. These facing opposite charges facilitated an electrostatic attraction between M and C_C≡N_. The extent of this electrostatic contribution can be assessed from the topological parameters of the M-C_C≡N_ BCP-s: the small electron density (around 0.05 au), positive Laplacian of electron density (around 0.12 au) and close to zero total electronic energy density (around −0.004 au, cf. Table S3) refer to a dominant ionic character of the M-(C≡NCy) interaction. These parameters were very close to the values of the M-C_Cp_ BCP-s (vide supra), supporting the comparable ionic character of the two metal–ligand interactions.

The (essentially) neutral character of the C≡NCy ligand means that the net CT to M^3+^ was marginal. The QTAIM charges of C_C≡N_ and N in the free ligand were computed to be +0.87 and −1.48 e, respectively. The interaction with M slightly decreased the C_C≡N_ and N atomic charges (cf. Table S3), making the C≡N bond less polarized.

**Figure 7:**
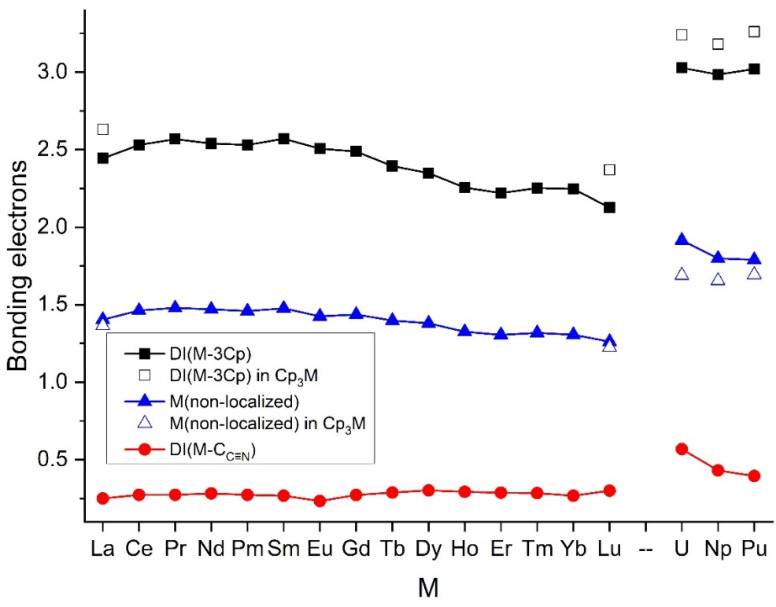
Delocalization indices between M and the contacting C atoms of the ligands as well as non-localized electrons on M in the (Cp)_3_M(C≡NCy) and selected (Cp)_3_M complexes.

The covalent interactions could be best assessed by the delocalization indices (DI), corresponding to the amount of electrons forming the orbital interaction between two atoms. The DI data depicted in Figure 7 indicated significant covalent interactions of M with both the Cp and C≡NCy ligands. The DI values summed over all the 15 M-C_Cp_ contacts in the M(Cp)_3_ moieties (black squares in Figure 7) were by one order of magnitude larger than the DI values between M and C_C≡N_. However, the DI value of a single M-C_Cp_ interaction was only ca. half of the DI of the M-C_C≡N_ bond. This points out that, in spite of the marginal CT, the M-(C≡NCy) covalent interaction was quite considerable.

The bonding properties of the (Cp)_3_An(C≡NCy) complexes showed characteristic differences compared to the Ln analogues, foreshown already by the above-discussed structural and spectroscopic features. Most significant was the larger covalent interaction of An^3+^ with both the Cp and C≡NCy ligands (Figure 7). This was based on the considerably larger non-localized electron density around An with respect to Ln, whereas the net CT from the ligands was not much larger (Table S3). The bonding characteristics of the three An included a gradually increasing charge transfer (from both ligands) from U to Pu. This resulted, in the first place, in decreasing the An charges in terms of the QTAIM. The DI and An non-localized electron values mostly decreased too (Figure 7). In fact, there were no large differences in the bonding of the three An^3+^ ions with the Cp ligands in terms of the DI. The less CT to U seemed to be compensated by the larger number of U valence (non-localized) electrons participating in the bonding with the Cp-s, resulting in the large DI value, as compared to Np and Pu.

In contrast, there was a significant difference in the interaction with the C≡NCy ligand. There was practically no net charge transfer in terms of the QTAIM from C≡NCy to An^3+^. Moreover, particularly the data of the U complex implied a significant U → C≡NCy back donation.

Regarding the DI data on the M-(C≡NCy) bonding, most significant was the value in the U complex, referring to a very strong covalent interaction compared to both the Ln and the other two An metals. This strong interaction incorporated the above-mentioned significant back donation from U to the C≡NCy ligand. The structural consequence of this interaction was the C≡N bond distance being the longest in the U complex compared to the other (Cp)_3_M(C≡NCy) molecules.

Because the QTAIM analysis deals with the global electron density distribution (i.e., the superimposed result of donations and back donations), it does not give information on the individual ligand-to-metal donation and metal-to-ligand back-donation interactions. To gain more insight into the above CT details, we applied the Natural Bond Orbital (NBO) analysis, which can estimate the individual charge transfer interactions. The NBO analyses were performed for the three An complexes, while from the Ln complexes for La and Lu. Selected NBO results are given in Table 3.

At the comparison of the natural atomic charges with the QTAIM ones, the different approaches used in the two models for the decomposition of the total electron density to the atoms should be taken into account. While the more sophisticated QTAIM operates by locating the atomic basins and integrating the electron density within them, the NBO approach is based on the simple Lewis model and assigns populations to the natural atomic orbitals.

Comparisons of the atomic charges in Table 3 and Table S3 reveal that the values of the natural atomic charges are considerably smaller and the relative order among the three An is opposite to the one by the QTAIM. Moreover, NBO predicted a significant net CT from the C≡NCy ligand to all M in contrast to the marginal ones by the QTAIM. These discrepancies indicate an overestimated assignment of the electron density between M and C_C≡N_ to the M atoms by NBO. On the other hand, the relative values of the NBO charge transfer and orbital population data between the various complexes were in qualitative agreement with the QTAIM results. Therefore, keeping in mind their qualitative character, we can use them for the discussion of the donation and back-donation interactions.

Thus, the CT energies from the NBO model described by the second-order perturbation energies (E_PT2_) in Table 3 were in agreement with the dominance of the 3Cp → M donation for all the metals. Only marginal M → Cp back donations were predicted for the present complexes. The acceptor orbitals in the 3Cp → M donation were mainly 5d orbitals with a marginal 4f contribution for La while with a minor 6s contribution for Lu. The An acceptor orbitals were mainly 6d orbitals with a slight 5f contribution.

**Figure 8:**
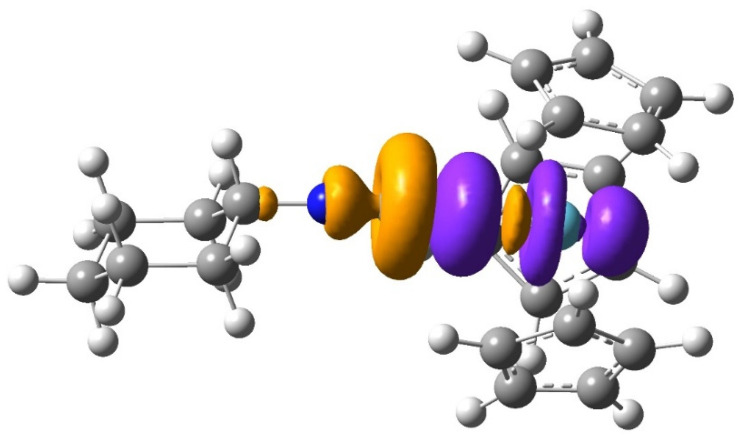
The NOCV pair (isovalue = 0.0004 au) demonstrating the σ-donation CyN≡C → 5d_0_ orbital of La in the (Cp)_3_La(C≡NCy) complex. The colors orange and violet mean the donated and accepted densities, respectively.

In the CT interactions with the C≡NCy ligand, both significant C≡NCy → M donations and An → C≡NCy back donations were indicated by the NBO analysis. The C≡NCy → M interactions were manifested in a major σ-donation from the C_C≡N_ lone pair to M. The main acceptor orbitals were the valence d_0_ orbitals of M, as presented in Figure 8 from ETS-NOCV (extended transition state-natural orbitals for chemical valence) analysis [56] of the (Cp)_3_La(C≡NCy) complex. The An → C≡NCy interactions corresponded to π back donations from the mixed An 5f orbitals to π*_C≡N_ orbitals. The Ln → C≡NCy back donations were not indicated by the E_PT2_ data, though some populations on the π*_C≡N_ orbitals were found in the La and Lu complexes too (cf. Table 3). These populations likely originated from intramolecular CT interactions within the C≡NCy ligand, the while back donation from Ln may have been hampered by the lack of appropriate Ln (4f) populated donor orbitals. The population of the σ*_C≡N_ orbital was 0.01 e in all the depicted complexes.

Being consistent with the CT and DI data in Figure 7, the E_PT2_ values in Table 3 support more favored CT interactions of the C≡NCy ligand with An over Ln. In addition, both the donation and back-donation interactions between An and C≡NCy decreased from U to Pu, in agreement with the DI values of the An-C_C≡N_ interaction. The trend in the back donation was further supported by the decreasing population of the π*_C≡N_ orbitals in this order (cf. Table 3).

The slightly decreasing covalent interactions from U to Pu agreed with the trend elucidated in topological studies of An(Cp)_3_ and An(Cp)_4_ complexes [25,26].

### 2.5. Competing Metal–Ligand Interactions: Cp_3_ vs. C≡NCy

In order to assess the effect of the C≡NCy ligand, representative M(Cp)_3_ complexes (M = La, Lu, U, Np, Pu) were computed in the present study at the same theoretical level as the title complexes. The bonding in these complexes was evaluated using the QTAIM model. Selected data are shown in Figure 2 and Figure 7 and are listed in Table S4.

In the isolated M(Cp)_3_ complexes, the Cp ligands surrounded M in a cylindrical arrangement [22,25,26,27]. The optimized structures possessed (or were close to) *C_3h_* symmetry at our computational level. Upon the steric effect of the C≡NCy ligand, the Cp groups were shifted towards the opposite side of M, forming a pyramidal arrangement. Consequently, the average M-C_Cp_ distances were increased by 0.02–0.04 Å depending on the ionic radii, where smaller radii caused a larger shift (cf. Figure 2). Accordingly, these larger M-Cp distances resulted in reduced DI values (by ca. 0.2 e) for the weakened M-(Cp)_3_ bonding (cf. Figure 7). On the other hand, the CT from the Cp ligands changed neither significantly nor uniformly for the five M. Noteworthy is the increased Cp → U CT upon introduction of the C≡NCy ligand in the U complex, compensating for the larger electron demand of the U-(C≡NCy) bonding (cf. its large DI in Figure 7).

The generally small effect of C≡NCy on the Cp → M CT and the above-noted marginal net CT between M and the C≡NCy ligand (in terms of the QTAIM) caused the M atomic charges to change only very slightly. In contrast, the non-localized electron densities on M, representing the contribution of M to the covalent bonding with both the Cp and C≡NCy ligands, were increased significantly, particularly on An. In other words, the C≡NCy ligand seemed to release some density from the non-bonding M atomic orbitals. This additional non-localized density supplemented those moved from the M-(Cp)_3_ interaction (vide supra the reduced M-(Cp)_3_ DI-s) to the covalent bonding with the C≡NCy ligand.

## 3. Materials and Methods

### 3.1. Syntheses

All syntheses were carried out under an Ar atmosphere and inert conditions by applying Schlenk techniques. All glassware was evacuated and heated under vacuum prior to its usage. All solvents were dried and degassed according to laboratory standards. C≡NCy was dried over Na.

The general procedure for the synthesis of (Cp)_3_Ln(C≡NCy) was as follows: Approximately 1 mmol of LnCp_3_ was emulgated in 30 mL of n-pentane; 1.1 mol equivalent of C≡NCy was added and the emulsion was stirred for 18 h at RT. The solvent was removed in vacuum and the residue was extracted with n-pentane (25 mL plus 0.01 mL of C≡NCy) for 2–3 weeks. Very few insoluble materials remained on the glass filter of the frit. Crystals suitable for X-ray diffraction were already formed after the first day and were taken out for analysis. After the extraction, the solvent was taken out and the collected crystals were dried briefly in vacuum. The isolated yields were close to quantitative.

The synthesis of (Cp)_3_La(C≡NCy)_2_ occurred as follows: A total of 0.5 g (ca. 1.5 mmol) of LaCp_3_ was placed in a Schlenk tube, 1 mL of C≡NCy was added and the mixture was heated in a water bath to ~70 °C until the LnCp_3_ was dissolved completely. The solution was allowed to cool to RT in the water bath under the formation of colorless crystals. The extraction of the crystals with n-pentane led to the formation of single crystals of the form (Cp)_3_La(C≡NCy)*(Cp)_3_La(C≡NCy)_2_ with the C≡NCy mono- and bis-adduct to La(Cp)_3_ present in the same crystal.

The synthesis of (Cp)_3_An(C≡NCy) (An = U, Np, Pu) occurred as follows: The An complexes were all prepared in the same manner, as the detailed procedure for (Cp)_3_Pu(C≡NCy) described. A total of 130 mg (0.30 mmol) of PuCp_3_ was emulgated in 20 mL of n-pentane; 42 μL (0.34 mmol) of C≡NCy were added and the emulsion was stirred for 18 h at RT. The solvent was removed in vacuum and the residue was extracted with n-pentane (25 mL plus 0.05 mL of C≡NCy) for 2 weeks. Very few insoluble materials remained on the glass filter of the frit. Crystals suitable for X-ray diffraction were taken out after the first day for analysis. After the extraction, the solvent was removed and the collected crystals were dried briefly in vacuum, yielding 147 mg (0.27 mmol, 90%) of isolated (Cp)_3_Pu(C≡NCy). The yields for (Cp)_3_U(C≡NCy) and (Cp)_3_Np(C≡NCy) were 95 and 90%, respectively.

Caution: Np and Pu are radioactive substances and have to be handled in licensed radiological facilities respecting all necessary safety and security regulations.

### 3.2. Single Crystal XRD

The single crystal structure determinations of the (Cp)_3_M(C≡NCy) complexes were performed on a Bruker SMART CCD 1000 (Siemens Analytical X-ray Instruments Inc., Karlsruhe, Germany) or on a Bruker APEX II Quazar (Bruker AXS Inc., Madison, WI, USA) with monochromated Mo Kα irradiation collecting at least one sphere of data [57,58]. Frames were collected at low temperatures with an appropriate irradiation time between 1 and 10 s per frame using a ω-scan (Bruker SMART CCD 1000, Δω = 0.45°) or a combined ω- and φ-scan technique (Bruker APEX II Quazar, Δω = Δφ = 0.5°). Data were integrated with SAINT and corrected to Lorentz and polarization effects; an experimental adsorption correction with SADABS was applied [57,58]. The structures were solved by direct methods and refined to an optimum R_1_ value with SHELX-2017 [59]. Visualization for evaluation was performed with winray-32 [60]. For more details, refer to Table S1 of the Supplementary Material.

The structures were deposited at The Cambridge Crystallographic Data Centre with the reference CCDC numbers 2,171,156–2,171,170, 2,172,154. They contain the supplementary crystallographic data for this paper. These data can be obtained free of charge from the CCDC via www.ccdc.cam.ac.uk/data_request/cif (accessed on 13 June 2022).

### 3.3. NMR Spectroscopy

The NMR spectra of a double encapsulated sample of (Cp)_3_Pu(C≡Ncy) in C_6_D_6_ were registered at RT on a Bruker Avance 400 NMR spectrometer (Karlsruhe, Germany) using standard Bruker pulse programs using gradient pulses. Under these conditions, the sample was stable enough for registration of the spectra. Shifts are given in ppm relative to the solvent standard.

### 3.4. Computational Details

The computations were performed with the Gaussian09 suit of programs [61] using the B3LYP [62,63] exchange-correlation functional. The B3LYP functional was extended with the D3 version of Grimme’s dispersion correction using the original D3 damping function [64]. Being important for weak interactions, the SuperFine grid was applied for integration accuracy. It contains 150 radial shells and 974 angular points per shell for C, N and H, and 225 radial shells for the lanthanides and actinides.

For the lanthanides, small-core 4f-in-valence quasi-relativistic pseudo-potentials [65] were applied. The valence basis set treating the 4s4p4d4f5s5p5d6s orbitals had a contraction scheme of [14s13p10d8f6g]/[10s8p5d4f3g] [66]. For the actinides, the small-core 5f-in-valence quasi-relativistic pseudo-potentials (ECP60MWB) [67,68] were utilized in conjunction with a [14s13p10d8f6g] valence basis set contracted to [10s9p5d4f3g] [68]. For C, N and H, the standard 6-311+G** basis set was used. The spin multiplicities of the model structures (given in Table S2) corresponded to the Aufbau (high spin) electron configurations of the M^3+^ ions. The possible lowest-energy electronic structure of the open-shell complexes was ensured by employing the Stable keyword of Gaussian09. The geometry optimizations were followed by frequency calculations, confirming the minimum characters of the obtained structures.

The topological analysis of the electron density distribution was based on the Quantum Theory of Atoms in Molecules (QTAIM) [69] utilizing the AIMAll code [70]. The natural atomic charges, valence orbital populations and second-order perturbation energies were evaluated on the basis of the Natural Bond Orbital (NBO) model [71] using the NBO 6.0 code [72,73]. Due to the deficiency of the NBO 6.0 code for g functions, in these model calculations the g polarization functions were omitted from the metal basis sets. For visualization purposes, the GaussView 5 [74] and Multiwfn 3.8 [75] softwares were applied.

## 4. Conclusions

In the present study, the structure and competing bonding interactions of f elements with Cp and C≡NCy ligands were elucidated by a joint experimental (XRD and NMR) and theoretical analysis. The conformation of (Cp)_3_M(C≡NCy) generally found in the crystal corresponded to the major one in a C_6_D_6_ solution of (Cp)_3_Pu(C≡NCy). The larger M^3+^ ions facilitated the coordination of a second C≡NCy ligand at the opposite site of M. The crystal unit cell of (Cp)_3_La(C≡NCy)_2_ consisted, in a unique way, of three different conformers of the complex.

The structural and bonding properties of the (Cp)_3_An(C≡NCy) complexes showed characteristic differences with respect to the Ln analogues. In the structural properties, they appeared in the longer C≡N bond lengths and in the different trend of the An-C_C≡N_ bonds. In agreement with these differences in the C≡N bond lengths, the C≡N stretching frequency in the An complexes was found to be lower than those of the (Cp)_3_Ln(C≡NCy) analogues. The above features (confirmed both experimentally and theoretically) indicated stronger covalent bonding with the C≡NCy ligand in the An complexes, with a trend of U > Np > Pu.

The metal–ligand interactions were analyzed using the QTAIM and NBO models. They indicated a significant CT from the anionic Cp ligands to the metals, particularly to An. In contrast, CT interactions with the neutral C≡NCy ligand were found to be non-significant. However, the delocalization indices (DI) indicated substantial bonding electron densities with C≡NCy too. Here, the general trend of An > Ln and U > Np ~ Pu was predicted. In the competition of the Cp and C≡NCy ligands, the latter one achieved larger specific (electrons/one M-C interaction) bonding densities with M.

## Figures and Tables

**Table 2:** Assignment of the ^1^H and ^13^C NMR resonance signals (ppm) of (Cp)_3_Pu(C≡NCy).

δ(^1^H)	Intensity	δ(^13^C)	Assignment ^1^
12.99	15H	75.0	Cp
3.56	1H	45.6	H_1a_
0.51	2H	21.4	H_3e_
0.23	2H	21.4	H_3a_
0.46	2H	23.2	H_4a_, H_4e_
0.01	2H	35.4	H_2e_
−0.29	2H	35.4	H_2a_

^1^ For the numbering, see Figure 5. H_3e_, H_4a_ and H_4e_ overlap in the ^1^H NMR spectrum.

**Table 3:** Selected result from the NBO analysis of (Cp)_3_M(C≡NCy) complexes ^1^.

M	Atomic Charge			Net CT from		Pop	E_PT2_^2^ from		
	M	C_C≡N_	N	3Cp	C≡N	π*_C≡N_	C_C≡N_	π_C≡N_	M
La	1.33	0.34	−0.45	1.47	0.20	0.05	759	20	0
Lu	1.48	0.28	−0.44	1.37	0.15	0.08	583	60	0
U	0.85	0.38	−0.45	1.93	0.22	0.19	1441	42	35
Np	0.95	0.37	−0.44	1.81	0.24	0.13	1070	34	28
Pu	1.04	0.37	−0.45	1.73	0.22	0.12	839	36	24

^1^ The natural atomic charges, transferred charges (CT) and π*_C≡N_ populations are given in e and the second-order perturbation energies (E_PT2_) are given in kJ/mol. The net charge transfers were derived from the summed atomic charges of the ligands. ^2^ Main second-order perturbation energies for the M–(C≡NCy) interaction: lone pair of C_C≡N_ → M and π_C≡N_ → M donations as well as M → π*_C≡N_ back donations.

## Data Availability

Not applicable.

## Supplementary Materials

The following are available online at https://www.mdpi.com/article/10.3390/molecules27123811/s1: Crystallographic details for all complexes (Table S1); computed data presented in Figure 2 and Figure 7 and additional theoretical data (Tables S2–S4); ^1^H and HH correlation NMR spectra of (Cp)_3_Pu(C≡NCy) measured in C_6_D_6_ (Figures S1–S3); Cartesian coordinates of the optimized structures of (Cp)_3_M(C≡NCy) (M = La Tb, Lu, U, Np and Pu) and (Cp)_3_La(C≡NCy)_2_.
