# Supplementary material for: Competing Metal–Ligand Interactions in Tris(cyclopentadienyl)-cyclohexylisonitrile Complexes of Trivalent Actinides and Lanthanides
Source: Molecules. 2022 Jun 14;27(12):3811. doi: 10.3390/molecules27123811 (PMC9228681; doi:10.3390/molecules27123811)
Supplement: Supplementary file 1 [file molecules-27-03811-s001.zip › molecules-1747368-supplementary.pdf]

Supplementary Material

Competing Metal–Ligand Interactions in Tris(cyclopentadienyl)-cyclohexylisonitrile Complexes of Trivalent Actinides and Lanthanides

List of Content:

Table S1. Crystallographic details for all complexes and selected XRD results on the $(Cp)_3La(C\equiv NCy)$*$(Cp)_3La(C\equiv NCy)_2$ sample.

Table S2. Selected computed data: the spinmultiplicities (m) used in the open-shell calculations, geometrical parameters (Å) and C≡N stretching frequencies ($_{C\equiv N}$, cm^{-1}).

Table S3. Table S3. Selected QTAIM data (au) of $(Cp)_3M(C\equiv NCy)$ complexes.

Table S4. Selected QTAIM data (au) of $M(Cp)_3$ complexes.

Figure S1. ^1H NMR spectrum of $(Cp)_3Pu(C\equiv NCy)$ measured in C_6D_6.

Figure S2. ^1H NMR spectrum of $(Cp)_3Pu(C\equiv NCy)$ in the range from 0.7 to -0.45 ppm.

Figure S3. 2D HH correlation NMR spectrum of $(Cp)_3Pu(C\equiv NCy)$ in the range from 3.6 to -0.57 ppm.

Cartesian coordinates of the optimized structures of $(Cp)_3M(C\equiv NCy)$ (M = La Tb, Lu, U, Np, Pu) and $(Cp)_3La(C\equiv NCy)_2$

Table S1. Crystallographic details for the complexes [(Cp)$_3$M(CNCy)]

Compound	La	Ce	Nd	Sm	Eu	Gd
Formula	$C_{22}H_{26}NLa$	$C_{22}H_{26}NCe$	$C_{22}H_{26}NNd$	$C_{22}H_{26}NSm$	$C_{22}H_{26}NEu$	$C_{22}H_{26}NGd$
CCDC No.	2171158	2171161		2171159	2171165	2171166
Formula weight / g/mol	443.34	444.56	448.68	454.79	456.40	461.69
T / K	173	123	103	103	123	123
Crystal system	triclinic	monoclinic	monoclinic	monoclinic	monoclinic	monoclinic
Space group (no)	P-1 (2)	C2/c (15)	$P2_1/n$ (14)	$P2_1/n$ (14)	$P2_1/n$ (14)	$P2_1/n$ (14)
Z	4	8	4	4	4	4
Unit cell parameter<br>a / Å<br>b / Å<br>c / Å<br>α / °<br>β / °<br>γ / °	 8.3671(14)<br>11.642(2)<br>21.645(4)<br>91.030(2)<br>90.243(2)<br>104.259(2)	 24.193(16)<br>8.166(5)<br>22.920(15)<br><br>119.399(15) 	 8.2644(4)<br>20.530(1)<br>12.2666(7)<br><br>105.012(1) 	 8.2017(5)<br>20.205(1)<br>12.1592(7)<br><br>103.522(1) 	 8.1917(9)<br>20.483(2)<br>12.258(1)<br><br>104.981(1) 	 8.1892(4)<br>20.4984(9)<br>12.2452(6)<br><br>105.192(1)
Volume / $Å^3$	2043.0(6)	3945(4)	2010.3(2)	1959.1(2)	1986.8(4)	1983.7(2)
Density (calc.)/ g/cm^{-3}	1.441	1.497	1.482	1.542	1.526	1.546
Abs. coeff. / mm^{-1}	2.093	2.310	2.584	2.999	3.159	3.345
F(000)	888	1784	900	908	912	916
Crystal size / mm	0.25<br>0.35<br>0.35	0.108<br>0.077<br>0.022	0.044<br>0.093<br>0.161	0.167<br>0.111<br>0.054	0.132<br>0.064<br>0.056	0.091<br>0.083<br>0.061
θ range / °	1.805 to 28.379	1.932 to 28.354	1.984 to 26.726	1.996 to 28.504	1.987 to 28.459	1.987 to 27.425
Index ranges	-11 ≤ h ≤ 10<br>-15 ≤ k ≤ 15<br>-28 ≤ l ≤ 28	-32 ≤ h ≤ 32<br>-10 ≤ k ≤ 10<br>-29 ≤ l ≤ 29	-10 ≤ h ≤ 10<br>-25 ≤ k ≤ 25<br>-15 ≤ l ≤ 15	-11 ≤ h ≤ 11<br>-27 ≤ k ≤ 26<br>-16 ≤ l ≤ 16	-10 ≤ h ≤ 10<br>-26 ≤ k ≤ 26<br>-16 ≤ l ≤ 16	-10 ≤ h ≤ 10<br>-26 ≤ k ≤ 26<br>-15 ≤ l ≤ 15
Reflections<br>collected<br>independent<br>R(int)<br>obs. (I>2σ)	 24596<br>9797<br>0.0512<br>7165	 20274<br>4262<br>0.1848<br>3601	 34907<br>4264<br>0.0272<br>3816	 35639<br>4681<br>0.0260<br>4317	 27843<br>4602<br>0.0866<br>3459	 35344<br>4516<br>0.0300<br>3954
Coverage / % (θ=25°)	99.8	94.7	99.9	100	99.3	100
Data/restraints/param.	9797/0/516	4262/222/298	4264/0/220	4681/0/220	4602/0/221	4516/0/221
Goof on F^2	1.141	0.968	1.527	1.042	1.191	0.991
R1 [I>2σ(I)]	0.0716	0.0644	0.0174	0.0159	0.0346	0.0164
wR2 (all data)	0.1526	0.1697	0.0351	0.0508	0.0684	0.0335
Largest peak/hole /$eÅ^{-3}$	1.892/-2.034	1.633/-1.022	0.283/-0.322	0.491/-0.424	1.446/-0.695	0.290/-0.300

Standard deviation in parentheses.

Table 1 continued:

Compound	Tb	Dy	Ho	Er	Tm	Yb
Formula	$C_{22}H_{26}NTb$	$C_{22}H_{26}NDy$	$C_{22}H_{26}NHo$	$C_{22}H_{26}NEr$	$C_{22}H_{26}NTm$	$C_{22}H_{26}NTm$
CCDC No.	2171169	2171164	2171160	2171157	2171162	2171156
Formula weight / g/mol	463.36	466.94	469.37	471.70	473.37	477.48
T / K	123	123	203	103	123	203
Crystal system	monoclinic	monoclinic	monoclinic	monoclinic	monoclinic	monoclinic
Space group	$P2_1/n$ (14)	$P2_1/n$ (14)	$P2_1/n$ (14)	$P2_1/n$ (14)	$P2_1/n$ (14)	$P2_1/n$ (14)
Z	4	4	4	4	4	4
Unit cell parameter						
a / Å	8.141(1)	8.1284(4)	8.1320(5)	8.1001(6)	8.0821(4)	8.0959(5)
b / Å	20.212(2)	20.214(1)	20.477(1)	20.209(1)	20.215(1)	20.427(1)
c / Å	12.132(1)	12.1301(6)	12.2175(7)	12.1052(9)	12.1022(6)	12.1800(5)
β / °	103.936(1)	103.966(1)	105.230(1)	104.082(1)	104.273(1)	105.412(1)
Volume / $Å^3$	1937.8(4)	1934.2(2)	1963.0(2)	1922.0(2)	1916.2(2)	1949.1(2)
Density (calc.)/ g/cm^{-3}	1.588	1.604	1.588	1.630	1.641	1.627
Abs. coeff. / mm^{-1}	3.651	3.865	4.032	4.368	4.632	4.800
F(000)	920	924	928	932	936	940
Crystal size / mm	0.081<br>0.077<br>0.057	0.075<br>0.064<br>0.048	0.154<br>0.124<br>0.084	0.045<br>0.048<br>0.202	0.114<br>0.088<br>0.068	0.45<br>0.40<br>0.40
θ range / °	2.002 to 28.310	2.002 to 27.884	1.989 to 28.557	2.006 to 28.520	2.007 to 28.470	1.994 to 29.294
Index ranges	$-10 \le h \le 10$<br>$-26 \le k \le 26$<br>$-15 \le l \le 16$	$-10 \le h \le 10$<br>$-25 \le k \le 25$<br>$-15 \le l \le 15$	$-10 \le h \le 10$<br>$-27 \le k \le 26$<br>$-16 \le l \le 16$	$-10 \le h \le 10$<br>$-26 \le k \le 26$<br>$-15 \le l \le 16$	$-10 \le h \le 10$<br>$-25 \le k \le 25$<br>$-15 \le l \le 16$	$-10 \le h \le 10$<br>$-26 \le k \le 26$<br>$-16 \le l \le 16$
Reflections						
collected	33304	34827	35840	34633	31181	22856
independent	4573	4517	4713	4602	4521	4763
R(int)	0.0456	0.0251	0.0194	0.0220	0.0246	0.0291
obs. (I>2σ)	3856	4073	4361	4222	4133	4215
Coverage / % (θ=25°)	100	100	100	100	100	100
Data/restraints/param.	4573/0/221	4517/0/221	4713/0/221	4602/0/220	4521/0/221	4763/0/220
Goof on F^2	1.176	1.131	1.095	1.000	1.103	1.120
R1 [I>2σ(I)]	0.0229	0.0144	0.0144	0.0132	0.0145	0.0192
wR2 (all data)	0.0423	0.0316	0.0318	0.0310	0.0312	0.0389
Largest peak/hole/$eÅ^{-3}$	0.643/-0.905	0.579/-0.407	0.34/-0.455	0.44/-0.509	0.53/-0.492	0.412/-0.473

Table 1 1: continued

Compound	Lu	Pu[a]	$(Cp)_3La(C≡NCy)_2$	$(Cp)_3La(C≡NCy)\cdot(Cp)_3La(C≡NCy)_2$
Formula	$C_{22}H_{26}NLu$	$C_{22}H_{26}NPu$	$C_{29}H_{37}N_2La$	$C_{51}H_{63}N_3La$
CCDC No.	2171168	2171170	2171167	2171162
Formula weight / g/mol	479.41	546.44	557.26	995.86
T / K	170	100(2)	173	173
Crystal system	monoclinic	monoclinic	monoclinic	monoclinic
Space group (no)	$P2_1/n$ (14)	$P2_1/n$ (14)	C2/c (15)	$P2_1/n$ (14)
Z	4	4	16	4
Unit cell parameter				
a / Å	8.0799(3)	8.2028(16)	15.8758(14)	15.0815(16)
b / Å	20.476(1)	20.700(4)	21.5600(19)	19.102(2)
c / Å	12.1948(8)	12.189(2)	31.189(3)	16.6006(18)
β / °	104.873(1)	109.81(3)	97.634(1)	96.655(2)
Volume / $Å^3$	1952.6(1)	1947.1(8)	10581(2)	4750(1)
Density (calc.)/ g/cm^{-3}	1.639	1.864	1.387	1.393
Abs. coeff. / mm^{-1}	5.084	3.387	1.632	1.809
F(000)	944	1036	4508	2016
Crystal size / mm	0.133<br>0.128<br>0.108	0.050<br>0.035<br>0.015	0.30<br>0.40<br>0.45	0.25<br>0.35<br>0.40
θ range / °	1.989 to 28.490	1.968 to 28.189	1.317 to 28.410	1.631 to 28.281
Index ranges	$-10 \le h \le 10$<br>$-27 \le k \le 27$<br>$-16 \le l \le 16$	$-10 \le h \le 3$<br>$-27 \le k \le 13$<br>$-10 \le l \le 15$	$-20 \le h \le 21$<br>$-28 \le k \le 28$<br>$-41 \le l \le 41$	$-19 \le h \le 19$<br>$-25 \le k \le 25$<br>$-22 \le l \le 21$
Reflections				
collected	34847	5268	64006	49445
independent	4693	3301	13088	11517
R(int)	0.0218	0.0667	0.1139	0.0274
obs. (I>2σ)	4236	1649	5733	8655
Coverage / % (θ=25°)	100	79.6	100	100
Data/restraints/param.	4693/0/220	3301/18/217	13088/6/587	11517/45/567
Goof on F^2	0.956	1.017	0.853	1.015
R1 [I>2σ(I)]	0.0144	0.0835	0.0488	0.0268
wR2 (all data)	0.0301	0.1798	0.1126	0.0614
Largest peak/hole/$eÅ^{-3}$	0.368/-0.435	4.626/-3.000	1.046/-1.461	0.540/-0.661

[a]The low quality crystal of $(Cp)_3Pu(C≡NCy)$ decomposed during the measurement.

Appendix to Table S1. Selected XRD results on the $(Cp)_3La(C≡NCy)$*$(Cp)_3La(C≡NCy)_2$ sample.

complex	M-$C_{C≡N}$	C≡N	M-$C_{Cp,av}$
$(Cp)_3La(C≡NCy)$ moiety	2.706(3)	1.143(3)	2.837
$(Cp)_3La(C≡NCy)_2$ moiety	2.825(5)	1.142(5)	2.870

Table S2. Selected computed data: the spinmultiplicities (m) used in the open-shell calculations, geometrical parameters (Å) and C≡N stretching frequencies ($_{C≡N}$, cm^{-1}).

M	m[a]	M-$C_{C≡N}$	C≡N[b]	M-$C_{Cp,av}$	$_{C≡N}$[b]
La	1	2.741	1.163	2.874	2263
La′	1	2.817	1.165	2.907	2248
Ce	2	2.693	1.163	2.843	2258
Pr	3	2.668	1.163	2.822	2259
Nd	4	2.631	1.163	2.808	2257
Pm	5	2.625	1.163	2.794	2260
Sm	6	2.611	1.163	2.783	2258
Eu	7	2.661	1.164	2.795	2255
Gd	8	2.593	1.163	2.794	2260
Tb	7	2.540	1.163	2.747	2261
Dy	6	2.504	1.162	2.754	2263
Ho	5	2.490	1.163	2.730	2257
Er	4	2.478	1.163	2.720	2258
Tm	3	2.468	1.163	2.712	2257
Yb	2	2.474	1.163	2.710	2256
Lu	1	2.422	1.163	2.699	2257
U	4	2.495	1.170	2.813	2183
Np	5	2.557	1.166	2.802	2222
Pu	6	2.556	1.165	2.783	2240

[a]Spinmultiplicities of the metals. La′ corresponds to the axial-axial conformer of $(Cp)_3La(C≡NCy)_2$.

[b]The computed bond distance in the free C≡NCy ligand is 1.170 Å, while the stretching frequency 2173 cm^{-1}.

Table S3. Selected QTAIM data (au) of $(Cp)_3M(C{\equiv}NCy)$ complexes.[a]

M	Atomic charge[b]			CT from		n-loc	DI		BCP at M-C_{CN}		
	M	$C_{C≡N}$	N	3Cp	C≡NCy	M	M-$C_{C≡N}$	M-3Cp	(r)	▽ (r)	$H(r_P)$
La	1.85	0.82	-1.44	1.12	0.03	1.40	0.25	2.44	0.036	0.092	-0.002
La′	1.88	0.83	-1.44	1.08	0.02	1.39	0.20	2.25	0.030	0.082	-0.001
Ce	1.81	0.82	-1.44	1.16	0.03	1.46	0.27	2.53	0.039	0.102	-0.003
Pr	1.78	0.82	-1.44	1.18	0.04	1.48	0.27	2.57	0.040	0.105	-0.003
Nd	1.78	0.82	-1.44	1.20	0.02	1.47	0.28	2.54	0.042	0.113	-0.003
Pm	1.78	0.82	-1.44	1.19	0.03	1.46	0.27	2.53	0.041	0.112	-0.003
Sm	1.74	0.82	-1.44	1.23	0.03	1.48	0.27	2.57	0.041	0.115	-0.003
Eu	1.65	0.82	-1.44	1.33	0.02	1.42	0.23	2.51	0.036	0.108	-0.001
Gd	1.70	0.82	-1.44	1.28	0.02	1.44	0.27	2.49	0.042	0.117	-0.003
Tb	1.83	0.81	-1.44	1.15	0.02	1.40	0.29	2.40	0.046	0.127	-0.005
Dy	1.83	0.81	-1.43	1.15	0.02	1.38	0.30	2.35	0.049	0.133	-0.006
Ho	1.87	0.80	-1.44	1.13	0.00	1.33	0.29	2.26	0.048	0.144	-0.006
Er	1.88	0.80	-1.44	1.11	0.01	1.31	0.29	2.22	0.049	0.146	-0.006
Tm	1.87	0.80	-1.44	1.12	0.01	1.32	0.28	2.25	0.049	0.148	-0.006
Yb	1.86	0.80	-1.44	1.14	0.00	1.31	0.27	2.25	0.047	0.147	-0.005
Lu	1.90	0.79	-1.44	1.10	0.00	1.26	0.30	2.13	0.053	0.157	-0.007
U	1.87	0.73	-1.44	1.21	-0.08	1.92	0.57	3.03	0.061	0.174	-0.011
Np	1.79	0.79	-1.44	1.22	-0.01	1.80	0.43	2.98	0.052	0.152	-0.007
Pu	1.72	0.81	-1.44	1.27	0.01	1.79	0.40	3.02	0.051	0.148	-0.006

[a]The abbreviations in the heading mean: CT – charge transfer; n-loc – non-localised electrons (participating in the covalent interactions of M); DI – delocalization indices between M and $C_{C≡N}$ and summed over the 15 M-C_{Cp} contacts; BCP – bond critical point; (r) - electron density; ▽ (r) - Laplacian of electron density; H(r) – total electronic energy density. La′ refers to the $(Cp)_3La(C{\equiv}NCy)_2$ complex with axial-axial orientations of the C≡NCy ligands.

[b]The charges of $C_{C≡N}$ and N in the free ligand are +0.87 and -1.48 e, respectively.

Table S4. Selected QTAIM data (au) of $M(Cp)_3$ complexes.[a]

M	Q(M)	CT(3Cp)	n-loc(M)	DI(M-3Cp)
La	1.85	1.15	1.37	2.63
Lu	1.91	1.09	1.22	2.37
U	1.83	1.17	1.69	3.24
Np	1.78	1.22	1.66	3.18
Pu	1.71	1.29	1.69	3.26

[a]The abbreviations in the heading mean: Q – atomic charge; CT – charge transfer from the 3 Cp ligands; n-loc – non-localised electrons; DI – delocalization indices summed over the 15 M-C_{Cp} contacts.

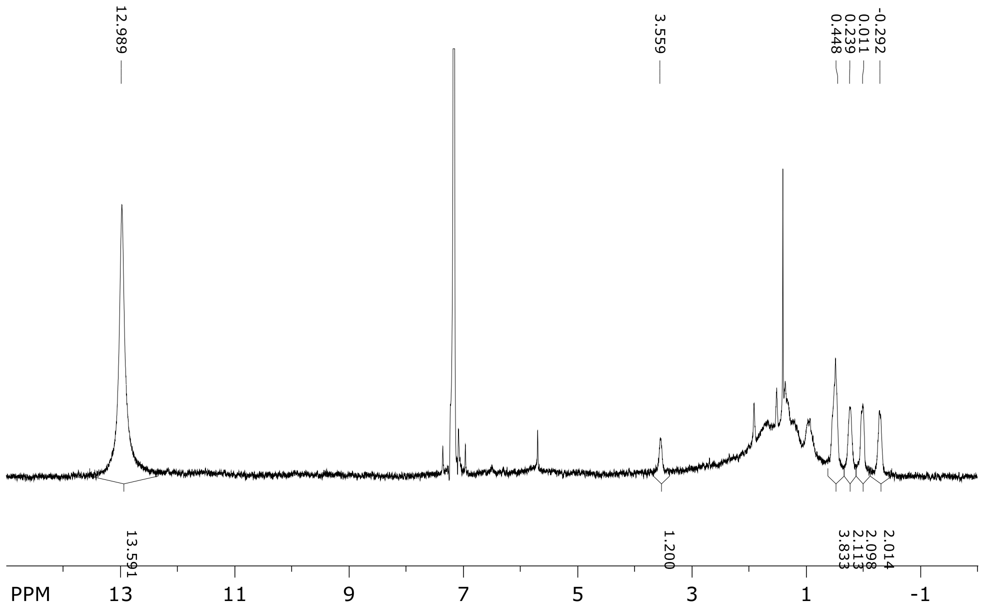

Figure S1. ^{1}H NMR spectrum of $(Cp)_3Pu(C{\equiv}NCy)$ measured in C_6D_6. Signal at 7.16 ppm is the residual proton signal of the solvent. Signals not marked with a peak picking do not arise from $(Cp)_3Pu(C{\equiv}NCy)$. The feature between 1 and 2 ppm is due to decomposition.

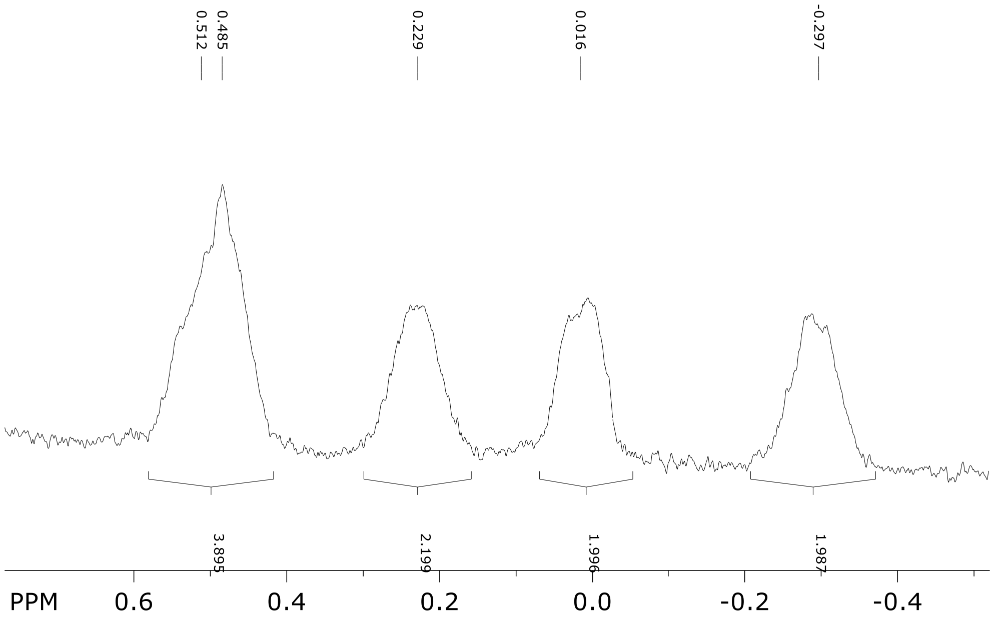

Figure S2. ^1H NMR spectrum of $(Cp)_3Pu(C{\equiv}NCy)$ measured in C_6D_6, in the range from 0.7 to -0.45 ppm representing the 10 protons of the five CH_2-groups of the cyclohexyl residue.

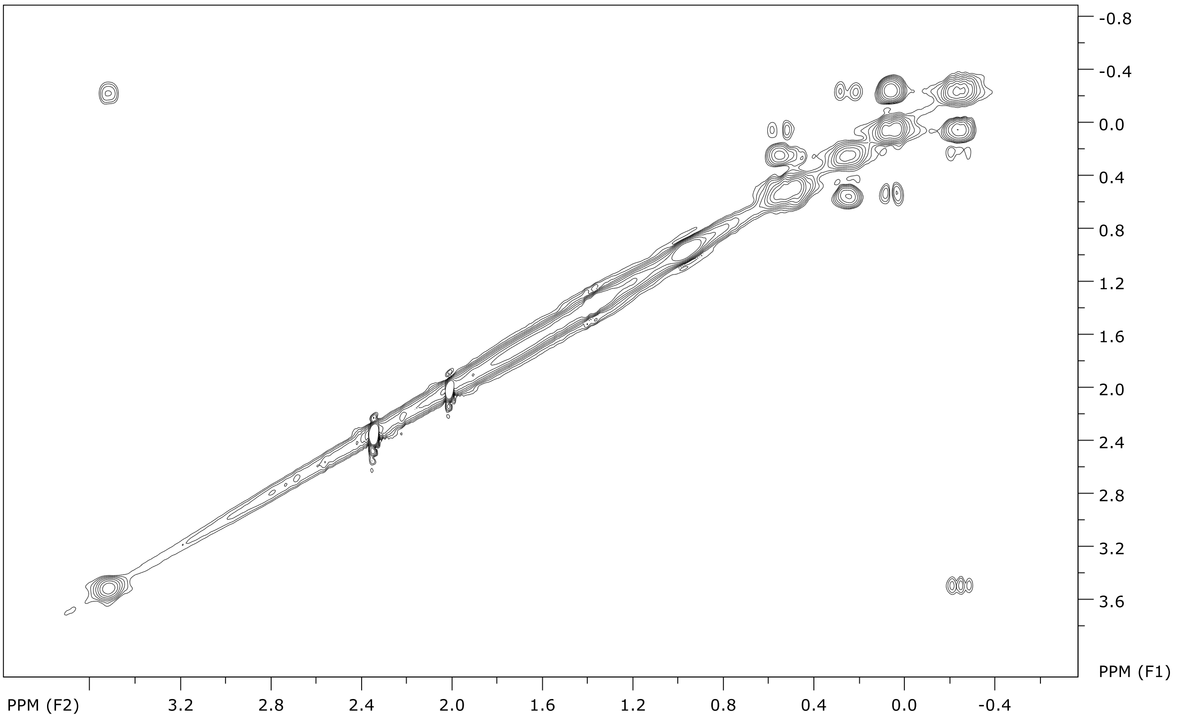

Figure S3. 2D HH correlation NMR spectrum of $(Cp)_3Pu(C{\equiv}NCy)$ measured in C_6D_6, in the range from 3.6 to -0.57 ppm, no other cross peaks are observed.

Cartesian coordinates of the optimized structures

(Cp)$_3$La(C≡NCy)

La	1.48296800	-0.00027700	-0.04918300
H	-7.58730400	-0.00056100	-0.31290500
H	-6.30236900	2.16238800	-0.27833700
C	-6.49944600	0.00114400	-0.42603300
H	-6.29435600	0.00427400	-1.50384200
H	-6.20159700	1.32284000	1.26034400
C	-5.90349200	1.26679800	0.20573100
H	-6.29675400	-2.16030300	-0.29011300
H	-6.19847600	-1.32887000	1.25316700
C	-5.90028100	-1.26635800	0.19894000
C	-4.37183700	1.27636000	0.10977100
H	-4.06104300	1.31152700	-0.94025300
H	-3.95166700	2.15668700	0.60290600
C	-4.36857800	-1.27153200	0.10324500
H	-4.05706300	-1.30119000	-0.94670400
H	-4.01393500	-0.00139200	1.81693300
C	-3.78901500	0.00153400	0.74454000
H	-3.94659900	-2.15313300	0.59258900
N	-2.35944400	0.00352600	0.62954500
C	-1.20740200	0.00401600	0.47349500
H	-0.68189600	2.57779600	-1.05436900
H	-0.18277300	-0.70309500	-3.02030900
H	-0.70264100	-2.53415000	-1.11755400
C	0.39314800	2.51981600	-0.95446900
H	0.22771600	-0.04601200	3.22630300
H	0.73336500	3.17424800	1.15439500
H	1.04377400	1.85166900	-2.97608900
C	0.55216400	-1.26312500	-2.45878500
C	0.27850000	-2.22448800	-1.45005500
C	1.14008700	2.84174200	0.20942900
C	1.30157100	2.14972400	-1.97032000
C	1.19215800	-0.29824300	2.80756300
H	0.97938800	-2.47244200	2.34053900
C	1.59053600	-1.58057000	2.34613500
H	2.30231500	1.62795900	2.92434700
C	1.95277900	-1.18251000	-2.61316700
C	2.28757600	0.58069100	2.65999000
C	1.51016000	-2.73379000	-0.98392100
H	2.47994200	-0.54962700	-3.31303600
C	2.50828900	2.67009700	-0.09158100
C	2.61004900	2.23045700	-1.43675600
H	1.63851200	-3.48342900	-0.21687100
C	2.54827700	-2.08321200	-1.69289200
H	3.33462100	2.84772700	0.58217500
H	3.52851000	2.03334200	-1.97354900
C	2.93159100	-1.48986400	1.91517200
C	3.36154200	-0.15014400	2.09784700
H	3.60797400	-2.27157200	-1.58272800
H	3.52902300	-2.29983100	1.52096100
H	4.35003900	0.23497600	1.88535300

(Cp)$_3$Tb(C≡NCy)

Tb	1.37925200	-0.00032200	-0.04009800
H	-7.49729500	-0.00136600	-0.32557500
H	-6.21213600	2.16151400	-0.30000900
C	-6.40976700	-0.00006500	-0.44200300
H	-6.20794400	0.00048800	-1.52044600
H	-6.10646700	1.32552300	1.24030700
C	-5.81170200	1.26704200	0.18488200
H	-6.20708800	-2.16122100	-0.30160900
H	-6.10376900	-1.32610300	1.23937500
C	-5.80883600	-1.26620600	0.18407300
C	-4.28038500	1.27595700	0.08416300
H	-3.97295700	1.30843700	-0.96698700
H	-3.85840300	2.15750400	0.57362400
C	-4.27745800	-1.27162800	0.08375200
H	-3.96912900	-1.30390600	-0.96710400
H	-3.91772300	0.00239500	1.79313000
C	-3.69520000	0.00275000	0.72010400
H	-3.85405500	-2.15216100	0.57387300
N	-2.26714200	0.00430800	0.59973700
C	-1.11676600	0.00437800	0.43113000
H	-0.75953200	2.47149100	-1.04595300
H	-0.22940800	-0.58852900	-2.90883600
H	-0.76814000	-2.45948600	-1.05350800
C	0.31243300	2.40926000	-0.93315700
H	0.07558100	-0.00991300	3.11290200
H	0.62537900	3.01051100	1.19380200
H	0.97906700	1.72167800	-2.93606400
C	0.49893500	-1.16740300	-2.36176500
C	0.21483000	-2.14727800	-1.37291300
C	1.04491700	2.70639700	0.24713000
C	1.22879100	2.03187600	-1.93418300
C	1.04197100	-0.26270500	2.70318800
H	0.81421200	-2.42182100	2.18435500
C	1.43571400	-1.54040100	2.22339100
H	2.14159300	1.66285400	2.81191000
C	1.89959500	-1.06957100	-2.48283900
C	2.13495800	0.61442600	2.56084200
C	1.43874000	-2.64893100	-0.88921200
H	2.43635300	-0.40368200	-3.13953300
C	2.41224500	2.51000100	-0.03229000
C	2.52802100	2.07121100	-1.37599600
H	1.55704500	-3.38699100	-0.11234000
C	2.48505600	-1.96968700	-1.55645800
H	3.22786800	2.63769100	0.66147300
H	3.44839800	1.83028300	-1.88717700
C	2.77217300	-1.44468500	1.78605600
C	3.20141100	-0.10606000	1.97367600
H	3.54316100	-2.12360700	-1.40417700
H	3.35843800	-2.23960300	1.35328400
H	4.17754500	0.28671700	1.73008000

(Cp)$_3$Lu(C≡NCy)

Lu	1.31422100	-0.00007900	-0.03636400
H	-7.44899900	-0.00093900	-0.32245600
H	-6.16204800	2.16091400	-0.30374500
C	-6.36182800	-0.00067500	-0.44239700
H	-6.16348700	-0.00162900	-1.52150100
H	-6.05183600	1.32668000	1.23723100
C	-5.76061500	1.26672500	0.18089300
H	-6.16059800	-2.16181400	-0.29994200
H	-6.05135100	-1.32477600	1.23960300
C	-5.75984000	-1.26654000	0.18324600
C	-4.22965400	1.27405500	0.07513000
H	-3.92574100	1.30488600	-0.97712100
H	-3.80520300	2.15594000	0.56188000
C	-4.22881600	-1.27323500	0.07794300
H	-3.92394600	-1.30714400	-0.97389800
H	-3.86291400	0.00247900	1.78439300
C	-3.64287100	0.00131200	0.71071600
H	-3.80451900	-2.15355200	0.56772700
N	-2.21610600	0.00168700	0.58548200
C	-1.06672300	0.00166200	0.40669400
H	-0.81975700	2.43289500	-1.06216000
H	-0.27607700	-0.57805000	-2.88637300
H	-0.81299300	-2.45033100	-1.02943900
C	0.24994800	2.35472300	-0.93423500
H	-0.00325800	0.00929300	3.08381200
H	0.54453800	2.97963700	1.19037400
H	0.93479800	1.68505400	-2.93562900
C	0.45388900	-1.13709600	-2.32009100
C	0.17004700	-2.12038900	-1.33200500
C	0.97211900	2.65302600	0.25420900
C	1.17528900	1.98031600	-1.92666400
C	0.95803700	-0.23936000	2.65854600
H	0.73746000	-2.40595200	2.16092300
C	1.35259900	-1.51871900	2.17875000
H	2.06189500	1.68123300	2.78691700
C	1.85462700	-1.03362500	-2.43451700
C	2.05157600	0.63630300	2.52046400
C	1.39344800	-2.61792300	-0.84561300
H	2.39313000	-0.38503400	-3.10770600
C	2.34182100	2.45784300	-0.01392400
C	2.46994600	2.01386000	-1.35559600
H	1.51070300	-3.36814700	-0.07987000
C	2.44079300	-1.92863400	-1.50250600
H	3.15507700	2.61273600	0.67785400
H	3.39760600	1.79575000	-1.86520500
C	2.69003700	-1.42229800	1.74462100
C	3.11798100	-0.08197900	1.92870700
H	3.49946300	-2.09417500	-1.36233700
H	3.28771800	-2.22317500	1.33797500
H	4.10256200	0.30492600	1.70791600

(Cp)$_3$U(C≡NCy)

U	1.24926800	0.00007900	-0.05018300
H	-7.57606500	-0.00002600	-0.31163500
H	-6.28921100	2.16148300	-0.28094000
C	-6.48772400	0.00005200	-0.42206600
H	-6.27992700	-0.00064400	-1.49948100
H	-6.19128300	1.32618400	1.26044800
C	-5.89112200	1.26707400	0.20647800
H	-6.28862600	-2.16107700	-0.27843800
H	-6.19138500	-1.32394200	1.26204200
C	-5.89087600	-1.26604300	0.20810000
C	-4.35939600	1.27317900	0.11393100
H	-4.04630600	1.30391700	-0.93577100
H	-3.93860700	2.15528100	0.60364800
C	-4.35909300	-1.27205900	0.11602800
H	-4.04498100	-1.30547500	-0.93324100
H	-4.01194000	0.00210300	1.82515800
C	-3.77426000	0.00115900	0.75447000
H	-3.93888200	-2.15304100	0.60833100
N	-2.34976100	0.00115800	0.64356500
C	-1.19533500	0.00113000	0.44827400
H	-0.92490000	2.51670400	-1.03491400
H	-0.63030800	-0.54899200	-2.88803200
H	-0.92949900	-2.50114900	-1.06143200
C	0.13940600	2.48037600	-0.85494400
H	-0.02496600	-0.06233200	3.17250200
H	0.29724400	3.02147900	1.30665800
H	0.96036200	1.95203400	-2.85271100
C	0.16704800	-1.10783200	-2.41938700
C	0.00946000	-2.13195700	-1.44689200
C	0.78582000	2.75851600	0.37944100
C	1.13293800	2.18687300	-1.81370100
C	0.91294300	-0.37454100	2.73748500
H	0.52629400	-2.50762100	2.19962500
C	1.20761700	-1.66963600	2.23336600
H	2.17068100	1.45098400	2.91274600
C	1.54646000	-0.95804600	-2.67359500
C	2.07093900	0.42174800	2.60408500
C	1.29201700	-2.60732300	-1.09928200
H	1.99411400	-0.27007700	-3.37551100
C	2.17699300	2.64141500	0.17782200
C	2.39675300	2.27048200	-1.17439600
H	1.50509500	-3.39826100	-0.39678400
C	2.24892300	-1.87204900	-1.84555900
H	2.94422000	2.80417700	0.92040000
H	3.35996100	2.13301200	-1.64765200
C	2.54862700	-1.67127000	1.79537400
C	3.08282600	-0.37350100	2.00711100
H	3.32025800	-2.02247000	-1.83051400
H	3.07976600	-2.51129000	1.37234500
H	4.09876000	-0.06281700	1.80221400

(Cp)$_3$Np(C≡NCy)

Np	1.23613800	0.00691300	-0.04044100
H	-7.63025600	0.04112400	-0.30243200
H	-6.36424400	2.19477600	-0.00509400
C	-6.54121900	0.06345200	-0.40208800
H	-6.32465400	0.19160600	-1.47003100
H	-6.27146600	1.18449000	1.42794900
C	-5.96222800	1.25245300	0.37718800
H	-6.32232700	-2.09780400	-0.51231700
H	-6.24646200	-1.44773200	1.11701700
C	-5.93788500	-1.26303900	0.08019500
C	-4.42987200	1.28328400	0.29980500
H	-4.10863600	1.44074600	-0.73583300
H	-4.02200600	2.10458600	0.89482200
C	-4.40539900	-1.24600600	0.00179200
H	-4.08245400	-1.15349300	-1.04107200
H	-4.07925800	-0.17838100	1.85283900
C	-3.84049200	-0.05106200	0.79066400
H	-3.98112300	-2.17495200	0.39157700
N	-2.41253400	-0.02748100	0.68922000
C	-1.26096800	-0.01100800	0.50891100
H	-0.86700800	2.73438100	-0.45952200
H	-0.70504000	-0.29507800	-2.83727800
H	-1.04090600	-2.36006300	-1.14205700
C	0.20358100	2.59903300	-0.50648200
H	0.08244100	-0.49792100	3.17348400
H	0.84686500	2.97586800	1.59727400
H	0.55122100	2.13147300	-2.65416200
C	0.07990700	-0.90829600	-2.41882100
C	-0.09683900	-1.99634900	-1.52069800
C	1.10987700	2.73624000	0.57704500
C	0.95246800	2.29579300	-1.66563000
C	1.02955300	-0.67883100	2.68590700
H	0.80656500	-2.76562300	1.93000300
C	1.41553400	-1.87998900	2.03812800
H	2.13931200	1.22404300	3.02465000
C	1.46095900	-0.79325900	-2.69113600
C	2.11509800	0.22533800	2.61545900
C	1.17127500	-2.54218700	-1.23752700
H	1.92089100	-0.07307700	-3.35138700
C	2.41612200	2.52199300	0.08396000
C	2.31972900	2.24089700	-1.30419000
H	1.37032100	-3.38097600	-0.58825600
C	2.14160100	-1.79123300	-1.94794600
H	3.32880900	2.57474000	0.66005500
H	3.14740000	2.05388100	-1.97423900
C	2.73980300	-1.71879200	1.57195400
C	3.16976100	-0.41274200	1.91920700
H	3.20655600	-1.97934100	-1.96182600
H	3.32466600	-2.46081800	1.04833900
H	4.14554500	0.00889700	1.71981800

(Cp)$_3$Pu(C≡NCy)

Pu	1.25135600	-0.00032700	-0.04393300
H	-7.63684500	-0.00141100	-0.32204300
H	-6.35241700	2.16173600	-0.29126400
C	-6.54891800	0.00022000	-0.43526500
H	-6.34389500	0.00151700	-1.51313700
H	-6.25051500	1.32455100	1.24872200
C	-5.95277000	1.26705900	0.19404200
H	-6.34633600	-2.16095900	-0.29565500
H	-6.24720700	-1.32660000	1.24608200
C	-5.94930500	-1.26618200	0.19160800
C	-4.42123500	1.27600500	0.09769100
H	-4.11078500	1.30917900	-0.95260100
H	-4.00058400	2.15743400	0.58858700
C	-4.41770800	-1.27079500	0.09567200
H	-4.10628900	-1.30232500	-0.95434500
H	-4.06371600	0.00183300	1.80653200
C	-3.83623200	0.00292700	0.73435700
H	-3.99525500	-2.15162700	0.58615800
N	-2.40908200	0.00483600	0.61735200
C	-1.25762700	0.00496300	0.44241600
H	-0.95696600	2.46774700	-1.02479200
H	-0.43597800	-0.61848000	-2.91981800
H	-0.96529600	-2.45673700	-1.02971900
C	0.11689200	2.42280600	-0.92130500
H	-0.10818000	-0.01161500	3.12578800
H	0.44135100	3.05062800	1.19529400
H	0.77995200	1.76262800	-2.93898600
C	0.29491100	-1.19026800	-2.36727500
C	0.01567100	-2.15679100	-1.36687100
C	0.85520500	2.73552500	0.24887900
C	1.03211400	2.06293500	-1.93372500
C	0.86236000	-0.26817700	2.72811300
H	0.64400200	-2.43344100	2.23521700
C	1.26076500	-1.54733400	2.26210900
H	1.97358300	1.65375500	2.86062100
C	1.69861800	-1.11934500	-2.52377400
C	1.96213700	0.60697900	2.59907900
C	1.24399500	-2.67307400	-0.90177200
H	2.22953000	-0.48665700	-3.21930000
C	2.22870600	2.57628700	-0.04788900
C	2.33881200	2.14946200	-1.39553200
H	1.36730900	-3.41957200	-0.13251600
C	2.28749300	-2.02866700	-1.60922600
H	3.05008200	2.75221300	0.63063300
H	3.25928800	1.95212800	-1.92732700
C	2.61213900	-1.46045300	1.85409200
C	3.04404900	-0.12455900	2.05216200
H	3.34558700	-2.21783400	-1.49226400
H	3.21173900	-2.26942000	1.46409500
H	4.03346900	0.26105300	1.84873500

(Cp)$_3$La(C≡NCy)$_2$ axial-axial

La	0.01729400	-0.56060100	-0.00320600
N	-3.87397100	-0.46166800	0.74620800
C	0.45248900	-2.11897100	-2.42879100
H	1.48378500	-2.30184800	-2.69325100
C	-0.32714200	-1.01094100	-2.84165900
H	0.00912500	-0.19484800	-3.46546000
C	-1.62198200	-1.16372300	-2.29798900
H	-2.45392600	-0.48713600	-2.43852000
C	-1.64239900	-2.37002200	-1.55003900
H	-2.49284000	-2.77845900	-1.02295400
C	-0.36118900	-2.95747300	-1.63199900
H	-0.05710200	-3.88455800	-1.16776000
C	-2.71023400	-0.49914400	0.71707200
C	-5.29908700	-0.32722400	0.60264200
H	-5.73503000	-1.27677700	0.92427100
C	-5.62770600	-0.07130900	-0.88256900
H	-6.71815100	-0.11434700	-0.98137800
H	-5.21259200	-0.87766900	-1.49250700
C	-5.11261000	1.29639900	-1.34905100
H	-4.01725700	1.29531000	-1.33289000
H	-5.40730400	1.46058100	-2.38928700
C	-5.64093600	2.42933600	-0.45628400
H	-6.72989300	2.50362200	-0.57488100
H	-5.22367800	3.38725400	-0.77946000
C	-5.31050600	2.18524600	1.02403100
H	-4.22713800	2.24128300	1.17361400
H	-5.74939100	2.96982900	1.64690900
C	-5.81467900	0.81581100	1.49744300
H	-6.90905500	0.78492800	1.45328000
H	-5.52708300	0.62759400	2.53515500
C	1.11432200	2.06662000	0.48907700
H	2.05424400	2.05319400	1.02339800
C	-0.16878300	2.13047400	1.07514500
H	-0.38465300	2.16998800	2.13337800
C	-1.12389100	2.14121600	0.02892900
H	-2.19418600	2.21286700	0.14919000
C	-0.43182200	2.08552300	-1.20207800
H	-0.88090700	2.07772700	-2.18472000
C	0.95062700	2.03822900	-0.92086100
H	1.74206400	2.00164200	-1.65573300
N	3.88130300	-0.43663900	-0.87433900
C	-0.36488900	-1.46747300	2.72618100
H	-1.29456300	-1.16309100	3.18500900
C	0.86129000	-0.76859400	2.79424500
H	1.03214400	0.16833900	3.30464700
C	1.82596900	-1.51294000	2.07890500
H	2.86966700	-1.25739700	1.96952200
C	1.19591400	-2.67545300	1.56658800
H	1.67153900	-3.45033700	0.98166800
C	-0.15817200	-2.64753600	1.96761200
H	-0.90420200	-3.39665400	1.74113400
C	2.73016600	-0.56768400	-0.75592900
C	5.29406800	-0.16558000	-0.86536500
H	5.76580700	-0.94794700	-1.46570900
C	5.81188900	-0.23074500	0.58585600
H	6.90459600	-0.16455400	0.53857000
H	5.56434400	-1.20335000	1.01908600
C	5.25538500	0.91478100	1.44154700
H	4.17352900	0.78786700	1.55693800

H	5.68345800	0.85897400	2.44630100
C	5.54694300	2.28219400	0.80608200
H	6.63124000	2.45482000	0.80163100
H	5.10443700	3.07910800	1.41054000
C	5.01439800	2.35784700	-0.63240500
H	3.92099400	2.31284100	-0.61915400
H	5.27897600	3.31632600	-1.08774200
C	5.56291200	1.21471500	-1.49653700
H	6.64972500	1.31120300	-1.59779000
H	5.14011500	1.24462500	-2.50421000

(Cp)$_3$La(C≡NCy)$_2$ axial-equatorial

La	0.17744700	0.17378400	-0.14558600
N	4.01888400	0.42514400	-1.08241300
N	-3.65781400	-0.25862400	0.80938200
C	1.79233200	1.50562400	1.86502500
H	2.61369000	2.09046800	1.47583900
C	1.83327600	0.13095800	2.21683200
H	2.69124200	-0.52271200	2.14201900
C	0.55683900	-0.23304300	2.69929300
H	0.26513900	-1.21245400	3.05113300
C	-0.27261100	0.91379100	2.64535100
H	-1.30353400	0.96573100	2.96387800
C	0.49152900	1.98633100	2.13101000
H	0.14183500	2.99567100	1.96880000
C	-0.02232500	2.84347900	-1.24296200
H	0.53632600	3.59091500	-0.69754200
C	-1.35870500	2.45871200	-0.99500800
H	-2.00518000	2.86245100	-0.22788100
C	-1.70716100	1.45775800	-1.93900600
H	-2.66761200	0.96931600	-2.02336200
C	-0.58633800	1.22607200	-2.76586900
H	-0.53535200	0.52160100	-3.58345700
C	0.45550300	2.08082000	-2.33665900
H	1.43816400	2.15416600	-2.77930100
C	1.46738100	-2.32387500	-0.97732500
H	2.54121400	-2.30508600	-1.09095100
C	0.76401700	-2.68288400	0.19471900
H	1.20518900	-2.95809400	1.14187200
C	-0.61763500	-2.61785800	-0.09083000
H	-1.41797100	-2.84811000	0.59788300
C	-0.76962200	-2.21871600	-1.44410800
H	-1.70671000	-2.08475000	-1.96685400
C	0.51941300	-2.03684900	-1.99131300
H	0.74528600	-1.74105600	-3.00599900
C	2.86487400	0.46506800	-0.93050500
C	5.44500200	0.23989700	-1.11942100
H	5.77825800	0.57177300	-2.10623400
C	5.76296000	-1.25740000	-0.93084900
H	6.83803000	-1.38024000	-1.10341300
H	5.24311200	-1.84093100	-1.69520900
C	5.40026600	-1.74276700	0.47876800
H	4.31329800	-1.70026300	0.60743500
H	5.68447600	-2.79333200	0.58574300
C	6.08088300	-0.88781300	1.55809700
H	7.16789900	-1.03110500	1.49965200
H	5.77294800	-1.22363300	2.55242000
C	5.75536300	0.60316000	1.38446000
H	4.68990500	0.76875800	1.57114100
H	6.29513900	1.20017400	2.12488000
C	6.10965100	1.09704200	-0.02436300
H	7.19189200	1.03099700	-0.18290300
H	5.82633900	2.14447500	-0.15728800
C	-2.51037700	-0.16215800	0.63358400
C	-5.08416400	-0.34568000	0.91214200
H	-5.31394500	-0.54304500	1.96538800
C	-5.60526000	-1.51368200	0.05650300
H	-5.14367500	-2.44665200	0.39003800
H	-5.28969100	-1.34805800	-0.97955500
C	-7.13565100	-1.59404000	0.13456400
H	-7.48995900	-2.40807200	-0.50395500

H	-7.43426700	-1.84854700	1.15954100
C	-7.79023200	-0.26623800	-0.27151000
H	-7.58063900	-0.06943100	-1.33036800
H	-8.87755700	-0.33554500	-0.17345200
C	-7.25487600	0.89781200	0.57392900
H	-7.55956900	0.75853200	1.61907100
H	-7.69312100	1.84430600	0.24540400
C	-5.72504900	0.99105400	0.49880700
H	-5.41102300	1.22153100	-0.52517200
H	-5.34723000	1.79142800	1.14023200

(Cp)$_3$La(C≡NCy)$_2$ equatorial-equatorial

La	-0.00263000	0.20225100	-0.02128600
N	3.91235700	0.12579800	0.66641800
C	-1.45980900	-0.21232200	2.43328300
H	-2.22827800	-0.97000000	2.36303600
C	-1.62763900	1.17432300	2.18056200
H	-2.54796500	1.66157200	1.89113500
C	-0.38605400	1.81194000	2.39466900
H	-0.18641900	2.86797000	2.28299800
C	0.54999000	0.82437300	2.77885700
H	1.58580800	0.99772900	3.03201100
C	-0.11386800	-0.42703500	2.80375500
H	0.33077300	-1.37707400	3.06453400
C	1.54628500	1.93435700	-1.76912200
H	2.58764000	1.67016700	-1.88592100
C	1.01014000	2.79304200	-0.77497600
H	1.56983800	3.28860400	0.00627900
C	-0.38164600	2.89456300	-0.99310800
H	-1.07628800	3.48008100	-0.40711500
C	-0.70586400	2.09811700	-2.11993000
H	-1.68892300	1.98071900	-2.55247500
C	0.48586000	1.50681200	-2.59807100
H	0.57105500	0.84499700	-3.44784100
C	2.75426400	0.17915800	0.55310700
C	5.34024200	0.01844600	0.70677000
H	5.66251200	0.45183400	1.66043300
C	5.96302200	0.82371800	-0.44718400
H	5.65754200	1.87035500	-0.37004800
H	5.56257500	0.43754700	-1.39112600
C	7.49156900	0.68998300	-0.42995400
H	7.91431100	1.24128800	-1.27447900
H	7.88710600	1.16023300	0.47939600
C	7.92811400	-0.78107300	-0.47351700
H	9.01852400	-0.85321700	-0.42338500
H	7.62656500	-1.21873800	-1.43340400
C	7.29079300	-1.58333100	0.66957900
H	7.67761400	-1.21851900	1.62969100
H	7.57149400	-2.63805400	0.60144600
C	5.76115600	-1.46086900	0.65873900
H	5.35374400	-1.90508800	-0.25616100
H	5.31759900	-1.99498100	1.50305600
N	-3.92305400	0.14713800	-0.66505400
C	-0.13275300	-2.72549500	0.14131600
H	-0.44391000	-3.12506700	1.09574400
C	1.19481800	-2.43894500	-0.24672300
H	2.07824900	-2.59770200	0.35515500
C	1.16676300	-1.93102100	-1.57147700
H	2.02445200	-1.62427900	-2.15436200
C	-0.17923100	-1.90478800	-1.99846100
H	-0.53462500	-1.57540000	-2.96466000
C	-0.98240600	-2.39592300	-0.93877300
H	-2.05517700	-2.52150400	-0.96202600
C	-2.76183900	0.18657700	-0.58190200
C	-5.35268800	0.05665000	-0.65699500
H	-5.70689700	0.56635000	-1.56022400
C	-5.91827400	0.77471400	0.58113000
H	-5.60494500	1.82188700	0.57114400
H	-5.48246600	0.31464500	1.47482900
C	-7.44773100	0.65601200	0.61776600
H	-7.82836400	1.14227400	1.52027000

H	-7.87500900	1.20017700	-0.23420700
C	-7.90010300	-0.80986600	0.56372800
H	-8.99245900	-0.86800100	0.55399000
H	-7.56458800	-1.32479000	1.47264000
C	-7.32031500	-1.52460100	-0.66485900
H	-7.74104600	-1.08086800	-1.57623000
H	-7.61141800	-2.57871300	-0.66896400
C	-5.79009900	-1.41770400	-0.70762000
H	-5.35280000	-1.93565200	0.15319000
H	-5.38721100	-1.88851800	-1.60812800
